# Calpain Inhibition Increases SMN Protein in Spinal Cord Motoneurons and Ameliorates the Spinal Muscular Atrophy Phenotype in Mice

**DOI:** 10.1007/s12035-018-1379-z

**Published:** 2018-10-16

**Authors:** Sandra de la Fuente, Alba Sansa, Ambika Periyakaruppiah, Ana Garcera, Rosa M. Soler

**Affiliations:** Unitat de Senyalització Neuronal, Department Medicina Experimental, Universitat de Lleida-IRBLleida, Rovira Roure 80, 25198 Lleida, Spain

**Keywords:** Motoneurons, Survival motor neuron, Calpain, Spinal muscular atrophy, Calpeptin

## Abstract

Spinal muscular atrophy (SMA), a leading genetic cause of infant death, is caused by the loss of *survival motor neuron 1* (*SMN1*) gene. SMA is characterized by the degeneration and loss of spinal cord motoneurons (MNs), muscular atrophy, and weakness. *SMN2* is the centromeric duplication of the *SMN* gene, whose numbers of copies determine the intracellular levels of SMN protein and define the disease onset and severity. It has been demonstrated that elevating SMN levels can be an important strategy in treating SMA and can be achieved by several mechanisms, including promotion of protein stability. SMN protein is a direct target of the calcium-dependent protease calpain and induces its proteolytic cleavage in muscle cells. In this study, we examined the involvement of calpain in SMN regulation on MNs. In vitro experiments showed that calpain activation induces SMN cleavage in CD1 and SMA mouse spinal cord MNs. Additionally, calpain 1 knockdown or inhibition increased SMN level and prevent neurite degeneration in these cells. We examined the effects of calpain inhibition on the phenotype of two severe SMA mouse models. Treatment with the calpain inhibitor, calpeptin, significantly improved the lifespan and motor function of these mice. Our observations show that calpain regulates SMN level in MNs and calpeptin administration improves SMA phenotype demonstrating the potential utility of calpain inhibitors in SMA therapy.

## Introduction

Spinal muscular atrophy (SMA) is an autosomal recessive neuromuscular disorder and the leading genetic cause of infant mortality [1, 2]. It is characterized by the degeneration of spinal cord motoneurons (MNs) resulting in muscular atrophy and weakness [3, 4]. SMA disease is caused by the homozygous disruption of the *survival motor neuron 1* (*SMN1*) gene, located in chromosome 5q3 (telomeric region), which is responsible for the production of survival motor neuron (SMN) protein [5]. In humans, a duplication of the *SMN* gene (*SMN2*) exists in the centromeric region. Most of the transcripts from *SMN2* lack exon 7 and produce a truncated unstable SMN protein isoform; only 10–20% of *SMN2* transcripts are correctly spliced and produce full-length SMN protein, which is unable to compensate for *SMN1* loss in SMA [6, 7]. Thus, the number of copies of *SMN2* determines intracellular SMN levels, which in turn define SMA disease onset and severity [8].

Currently available therapies for SMA have demonstrated that elevating SMN protein ameliorates the disease symptoms [9]. Several mechanisms increase SMN levels, including *SMN2* expression and regulation of SMN protein stability [9–11]. The mechanisms involved in regulating SMN protein stability include the ubiquitin proteasome system (UPS) [12, 13], SMN oligomerization [13], histone deacetylase inhibitors [14], and autophagy [15, 16]. Some of the SMN stabilizers have been used to analyze their effect on SMA mice phenotype; recent results have shown that treatment of severe delta7 SMA mice with proteasome inhibitors and/or histone deacetylase inhibitors improves survival and motor function [11, 12, 17].

Calpains are a family of calcium-dependent proteases that participate in Ca^2+^-mediated signaling pathways involved in several cell processes. In humans, the best characterized calpain isoforms are μ-calpain (calpain 1), predominant in the central nervous system, and m-calpain (calpain 2), ubiquitous in all tissues [18]. Increases of cytosolic calcium in neurons activate calpains. Although the physiological roles of calpain have not yet been fully elucidated, pathological conditions often result in its overactivation. Calpains have been related to degradation of various cellular proteins in pathophysiological responses associated with Alzheimer disease, amyotrophic lateral sclerosis, Huntington disease, Parkinson disease, spinal cord injury, and ischemia [19–23]. Evidence of calpain activity in modulating SMN levels in muscle cells [24, 25] led us to explore a potential role of calpain in regulating SMN stability in MNs. We recently reported that endogenous reduction of calpain 1 increased autophagy and survival motor neuron (Smn) protein level in cultured mouse embryonic MNs [15]. In the present work, we further analyzed the effect of calpain inhibition on MNs from CD1 and two SMA mouse models. Our results confirmed our hypothesis that calpain reduction increases Smn level in cultured mouse MNs. Calpain 1 knockdown inhibited Smn reduction in culture conditions that increase intracellular calcium level and prevented neurite degeneration in vitro in Smn-reduced MNs from a mouse model of severe SMA. To explore the therapeutic potential of calpain inhibition to modify the SMA phenotype, we administered calpeptin (a calpain inhibitor) to postnatal animals from two severe SMA mouse models. Calpeptin treatment significantly extended survival and improved motor function. Our findings suggest that SMN protein can be regulated by calpain in spinal cord MNs and that calpeptin may be useful for the treatment of SMA.

## Materials and Methods

### Spinal Cord MN Isolation and Culture

MN cultures were prepared from embryonic 12.5-day (E12.5) CD1 or SMA mouse spinal cord essentially as previously described [26, 27]. Briefly, isolated cells were pooled in a tube containing culture medium. Isolated MNs were plated either using 15-mm glass coverslips placed in four-well tissue culture dishes for immunofluorescence (15,000 cells/well) or in four-well tissue culture dishes (Nunc, Thermo Fisher Scientific, Madrid, Spain) for survival experiments (15,000 cells/well), neurite degeneration evaluation (10,000 cells/well), and western blot analysis (50,000 cells/well). Wells and glass coverslips were coated with polyornithine/laminin (Sigma) as described [28]. Culture medium was Neurobasal medium (Gibco, Invitrogen, Paisley, UK) supplemented with B27 (2% *v*/*v*, Gibco), horse serum (2% *v*/*v*, Fisher Scientific), L-glutamine (0.5 mM, Gibco), and 2-mercaptoethanol (25 μM, Sigma) and a cocktail of recombinant neurotrophic factors (NTFs) (1 ng/ml brain-derived neurotrophic factor, 10 ng/ml glial cell line-derived neurotrophic factor, 10 ng/ml ciliary neurotrophic factor, 10 ng/ml cardiotrophin-1, and 10 ng/ml hepatocyte growth factor; Gibco). Twenty hours after plating, 2 μg/ml of aphidicolin (Sigma) was added to the culture medium and was maintained throughout the experiment.

### SMA Animals

SMA mice FVB·Cg-Tg (SMN2)^89Ahmb^Smn1^tm1Msd^/J and FVB.Cg-*Grm7*^*Tg(SMN2)89Ahmb*^Smn1^tm1Msd^ Tg(SMN2*delta7)4299Ahmb/J were kindly provided by Dr. Josep E Esquerda and Dr. Jordi Caldero (Universitat de Lleida-IRBLLEIDA). Heterozygous animals were crossed to obtain homozygous Smn^−/−^, SMN2^+/+^ (mutSMA) or Smn^−/−^, and SMN2delta7^+/+^ (SMNdelta7). Littermates mutSMA/SMNdelta7 and Smn^+/+^, SMN2^+/+^/Smn^+/+^, and SMN2delta7^+/+^ (WT) were used for the experiments. For MN purification, E12.5 embryos were removed from the uterus and a piece was snipped from the head for genotyping; for in vivo treatment, neonatal offspring were tattooed and a fragment of the tail was collected. The REDExtract-N-Amp Tissue PCR Kit (Sigma, St Louis, MO, USA) was used for genomic DNA extraction and PCR setup, with the following primers: WT forward 5′-CTCCGGGATATTGGGATTG-3′, SMA reverse 5′-GGTAACGCCAGGGTTTTCC-3′, and WT reverse 5′-TTTCTTCTGGCTGTGCCTTT-3′.

All procedures were in accordance with the Spanish Council on Animal Care guidelines and approved by the University of Lleida Advisory Committee on Animal Services.

### Plasmids and Production of Lentiviral Particles

For RNA interference experiments [26], constructs were generated in pSUPER.retro.puro (Oligo-Engine, Seattle, WA, USA) using specific oligonucleotides (Invitrogen) targeting the calpain 1 (shCalp1) sequence, indicated by capital letters as follows, forward, gatccccGCGCCAAGCAGGTAACTTAttcaagagaTAAGTTACCTGCTTGGCGCttttt, and reverse, agctaaaaaGCGCCAAGCAGGTAACTTAtctcttgaaTAAGTTACCTGCTTGGCGCggg. pLVTHM, pSPAX2, and pMD2G were kindly provided by Dr. Trono (University of Geneva, Switzerland). Viruses at 4 × 10^5^–1 × 10^6^ TU/ml were used for the experiments. Empty vector (EV) was used as a control. For lentiviral transduction, MNs were plated in four-well dishes. Medium containing lentivirus (2 TU/cell) was added 3 h later, and then changed after 20 h. In each experiment, green fluorescent protein (GFP)-positive cells were counted directly to monitor infection efficiency. Frequency of infection rose 99%.

### MN Survival and Neurite Degeneration Analysis

Evaluation of MN survival was performed as described previously [29]. Cells were plated in complete medium containing a cocktail of recombinant NTFs or in basal medium with no supplements or in complete medium containing the experimental conditions. Three days after treatment, large phase-bright neurons with long neurite processes present in photomicrographs of different microscopic areas of tissue-culture dishes (four central areas per well, three wells for each condition per experiment) were counted. The number of cells present in each dish on day 0 was considered our initial 100%. Counts were performed every 3 days in the same microscopic areas as the initial count. Survival was expressed as the percentage of cells counted with respect to the initial value (100%).

Morphometric analysis of neurite degeneration was performed as described [30], with modifications. Briefly, dissociated MNs were cultured as described above and phase-contrast microscopy images were obtained with a 40× lens at 6, 9, and 12 days after plating. A grid was created over each image with NIH ImageJ software [31], using the grid plugin (line area = 50.000). The cell-counting plugin was used to score each neurite. Degenerating and healthy cells were counted in at least 10 high-power fields per image (30–50 neurites) for each well. Three different wells were counted for each condition (with the observer blinded to the condition) and the experiments were repeated at least three times. Neurite segments were considered degenerated if they showed evidence of swelling and/or blebbing.

### Calpeptin In Vivo Administration

Mice were individually housed in propylene cages (33 cm × 18 cm × 14 cm) at an ambient temperature of 22 ± 2 °C and relative humidity of 40% ± 10%. Breeder mice were provided with ad libitum water and rodent chow. Mice were maintained on a 12 h/12 h light/dark cycle (light period 07:30 until 19:30). mutSMA/SMNdelta7 and WT littermates were assigned randomly to receive treatment or vehicle. Calpeptin (Calbiochem, Merk, Madrid, Spain) was dissolved in DMSO (50 mM calpeptin) and injected at a dose of 6 μg per gram of weight in saline solution. Vehicle groups received equal volumes of saline solution with the same amount of DMSO. Administration was via subcutaneous injection (SC, interscapular region) once a day starting from P0 to death with a prolypropilene sterile syringe (icogamma plus, 1 mL) and with a 30G needle (BD Mircolance). WT animals received treatment or vehicle to a maximum of 3–4 weeks. Birth was defined as postnatal day 0 (P0) for the experiments. Survival of animals as well as body mass, size, and behavior test (righting reflex and tube test) were analyzed.

### Behavior Analysis

All tests were conducted during the light period between 09:00 and 12:00 and before the administration. For the righting reflex test, each pup was turned onto its back on a flat surface and the time it took to stably place all four paws on the ground was recorded (cutoff time of 30 s). On the tube test, animals were placed head down, hanging by the hind limbs in a plastic 50-ml centrifuge tube with a cotton ball cushion at the bottom to protect the animal’s head upon its fall. Latency to fall from the edge of the tube was assessed over a 30-s period [32]. Three repetitions were performed for each test with a minimum of 2 min of rest between repetitions. Behavior tests were performed daily starting at P1 until P10.

### Western Blot Analysis

Western blots were performed as previously described [26]. Total cell lysates were resolved in SDS polyacrylamide gels and transferred onto polyvinylidene difluoride Immobilon-P transfer membrane filters (Millipore, Billerica, MA, USA) using an Amersham Biosciences semidry Trans-Blot (Buckinghamshire, UK). The membranes were blotted with anti-SMN (Clone 8) antibody (1:5000; BD Biosciences), anti-SMN1 (2F1) antibody (1:1000; Cell Signaling Technology), anti-Calpain1 antibody (1:1000; Biomol International Inc., Exeter, UK), and anti-Fodrin clone AA6 (1:4000; Biomol). To control the specific protein content per lane, membranes were reprobed with monoclonal anti-α-tubulin antibody (1: 50000; Sigma). Blots were developed using Luminata™ ForteWestern HRP Substrate (Millipore).

### Immunofluorescence

Cultured MNs were plated on glass coverslips and treated with lentivirus containing EV or shCalp1. Six days after lentiviral transduction, cells were fixed with 4% paraformaldehyde (Sigma) for 10 min and with methanol (Sigma) for an additional 10 min. MNs were permeabilized with 0.2% Triton X-100 and incubated for 1 h with 5% BSA in PBS. Primary antibody (SMN antibody, 1:100) was diluted in PBS and incubated overnight. After washing, the secondary antibody anti-mouse ALEXA555 (Invitrogen) was added at 1:400 dilution. Hoechst (1:400, Sigma) staining was performed to identify nuclear localization in MNs soma. Samples were mounted using Mowiol (Calbiochem) mounting medium. Microscopy observations were performed in a FV1000 Olympus confocal microscope (Tokyo, Japan).

### Statistical Analysis

All experiments were performed at least three times. Values were expressed as mean ± SEM. Statistical analyses were performed using GraphPad Prism (version 5) (GraphPad Software Inc.) Differences were assessed by log-rank Mantel-Cox test for survival comparisons, and by Student *t* test or two-way ANOVA with Bonferroni post hoc test for all other analysis; *P* values < 0.05 were considered significant.

## Results

### Calpain 1 Knockdown Increases Smn Protein Level in Cultured Spinal Cord MNs

**Fig. 1:**
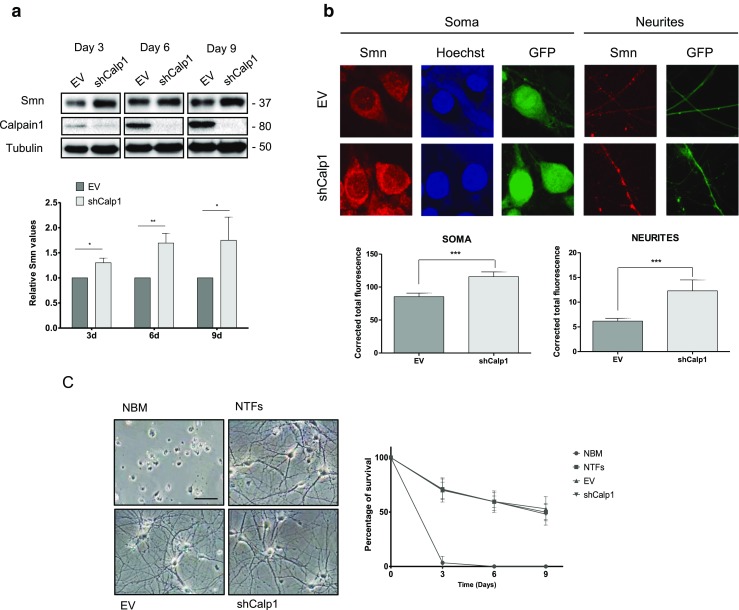
Effect of endogenous calpain reduction on Smn protein level and survival of cultured spinal cord MNs. Mouse MNs were transduced with lentivirus containing the shCalp1 or empty vector (EV) constructs and maintained in the presence of neurotrophic factors cocktail (NTFs). **a** Protein extracts of 3-, 6-, and 9-day transduced cultures were submitted to western blot analysis and probed with anti-SMN or anti-calpain antibodies. Membranes were reprobed with an anti-α-tubulin antibody, used as a loading control. Graph represents the expression of Smn and corresponds to the quantification of at least five independent experiments ± SEM. Asterisks indicate significant differences using Student *t* test (*p < 0.05; **p < 0.005). **b** Representative confocal images of 6-day EV- and shCalp1-transduced cells maintained in the presence of NTFs. Cells were fixed and immunofluorescence was performed with an anti-Smn antibody (red). Blue indicates Hoechst nuclear staining and green indicates green fluorescent protein (GFP) expression. Graphs represent the mean of relative Smn fluorescence (red) measured in soma (left) or neurites (right) of 6-day transduced cultures, corresponding to the quantification of five independent experiments ± SEM. Scale bar, 5 μm. Asterisks indicate significant differences using Student *t* test (SOMA *p* < 0.001; NEURITES *p* < 0.0005). **c** Representative phase-contrast images of non-transduced MNs cultured (6 days) in the presence or absence of NTFs (NBM, NTFs, respectively; top panels), and representative images of 6-day EV- and shCalp1-transduced cells (bottom panels). Graph represents the percentage of surviving cells after 3, 6, or 9 days in the following culture conditions: NBM, NTFs, EV- or shCalp1-transduced cells. The mean ± SEM corresponds to the quantification of five independent experiments. Scale bar, 30 μm

Calpains are involved in several muscle and neurodegenerative disorders, including SMA, and Smn is a direct target of calpain cleavage in muscle tissue [24, 25]. To analyze the effect of calpain reduction on Smn in spinal cord MNs, we used a lentiviral RNA interference method to downregulate the calpain protein level in these cells. Embryonic (E12.5) spinal cord MNs were isolated and plated in culture wells. Three hours later, we transduced them with lentivirus containing EV or short hairpin RNA sequences targeting specific sites of mouse calpain 1 (shCalp1) [26] and maintained in the presence of the neurotrophic factors cocktail (NTFs) (1 ng/ml brain-derived neurotrophic factor; 10 ng/ml glial cell line-derived neurotrophic factor, 10 ng/ml ciliary neurotrophic factor, 10 ng/ml cardiotrophin-1 and 10 ng/ml hepatocyte growth factor). At 3, 6, and 9 days after transduction, cell lysates were collected and submitted to western blot using an anti-SMN antibody. We observed a significantly increased Smn protein level in shCalp1 cells after 3 (1.30 ± 0.09, *p* < 0.05), 6 (1.69 ± 0.19, *p* < 0.005), and 9 (1.75 ± 0.46, *p* < 0.05) days of transduction, compared with the EV (Fig. 1a). Control blot analysis using an anti-calpain antibody demonstrated that calpain protein was reduced in shCalp1 condition compared to EV. To analyze whether this Smn reduction is preferentially localized in cell soma or neurites, we measured Smn levels with immunofluorescence using confocal microscopy. MNs were plated on glass coverslips and transduced using the EV or shCalp1 constructs. Six days after transduction, cells were fixed and Smn immunostaining with anti-SMN antibody was performed. Using the NIH ImageJ software, quantification of the average of fluorescence units (AFU) showed that Smn increases in both MN soma (AFU 115.80 ± 7.27, *p* < 0.001) and neurites (AFU 12.29 ± 2.21, *p* < 0.0005) in shCalp1 cells, compared to EV (AFU 85.43 ± 5.34 and 6.18 ± 0.56, respectively) (Fig. 1b).

To elucidate the effect of calpain reduction on MNs viability, survival experiments were performed. Cells were plated at low density to avoid cell contact-mediated effects (~ 8000 cells/cm^2^) and were cultured under several conditions: non-transduced cells in the absence (NBM) or the presence of NTFs, and EV- or shCalp1-transduced cells in the presence of NTFs. At 3, 6, and 9 days after plating, cells were counted in three wells of each condition. Considering 100% the initial number of cells present in a well on day 0, survival was estimated as the percentage of remaining cells in the same well. As shown in Fig. 1c, using the phase-contrast microscope, we observed that after 9 days without NTFs, MNs were largely degenerated and had a significantly lower percentage of surviving cells (day 3, NBM 3.38 ± 5.86% vs. NTFs 64.38 ± 3.90%, *p* < 0.001). However, the presence of EV or shCalp1 did not significantly modify MN survival, compared to the NTFs condition (day 9, 40.76 ± 2.63%, 44.11 ± 5.12%, and 42.35 ± 2.51%, respectively). Therefore, endogenous calpain reduction did not reduce MNs viability.

### Calpain 1 Knockdown Prevents Smn Reduction Caused by Membrane Depolarization

**Fig. 2:**
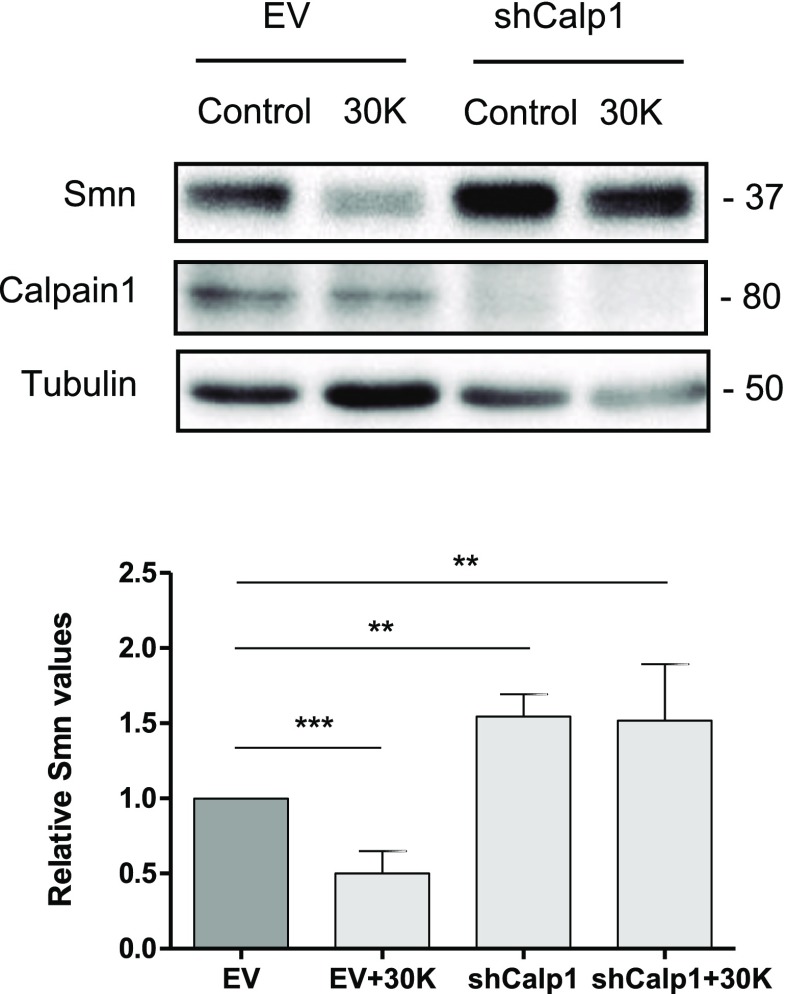
Calpain 1 knockdown prevents Smn reduction caused by membrane depolarization. Mouse MNs were transduced with lentivirus containing the shCalp1 or empty vector (EV) constructs and maintained in the presence of NTFs cocktail. Six days after transduction, cells were treated using basal medium with (30 K) or without (Control) 30 mM KCl during 3 h. Protein extracts were submitted to western blot using anti-SMN or anti-calpain antibodies. Membranes were reprobed with an anti-α-tubulin antibody. Graph values represent the expression of Smn vs α-tubulin and correspond to the quantification of three independent experiments ± SEM. Asterisks indicate significant differences using Student *t* test (**p < 0.005; ***p < 0.0001)

Addition of high K^+^ to the culture medium induces chronic depolarization of the neuronal plasma membrane, which in turn activates voltage-gated calcium channels (VGCCs), resulting in Ca^2+^ influx and elevation of intracellular Ca^2+^ concentration [26, 28]. Reaching the appropriate intracellular Ca^2+^ level induces the activation of Ca^2+^-dependent proteases such as calpains. To elucidate the effect of intracellular Ca^2+^ increase on Smn level and the mediating role of calpain, we isolated E12.5 mice spinal cord MNs and established a primary culture in the presence of NTFs. Cells were transduced with EV or shCalp1 constructs. Six days later, cells were treated with 30 mM of KCl (30K) for 3 h and protein extracts were obtained and submitted to western blot using anti-SMN antibody. Smn protein was significantly reduced in 30K EV-treated cultures (0.5 ± 0.15-fold, *p* < 0.0001) compared to non-treated EV control (Fig. 2). As expected, shCalp1 (1.5 ± 0.15-fold) treatment induces Smn increase when compared with EV control (*p* < 0.005). However, no significant differences of Smn level were observed in 30K shCalp1, compared to shCalp1 condition (1.5 ± 0.4- and 1.5 ± 0.15-fold, respectively), suggesting that high K^+^ treatment induced Smn protein reduction through calpain activity in cultured spinal cord MNs.

### Calpeptin Treatment Increases Smn Protein Level in Cultured Spinal Cord MNs

**Fig. 3:**
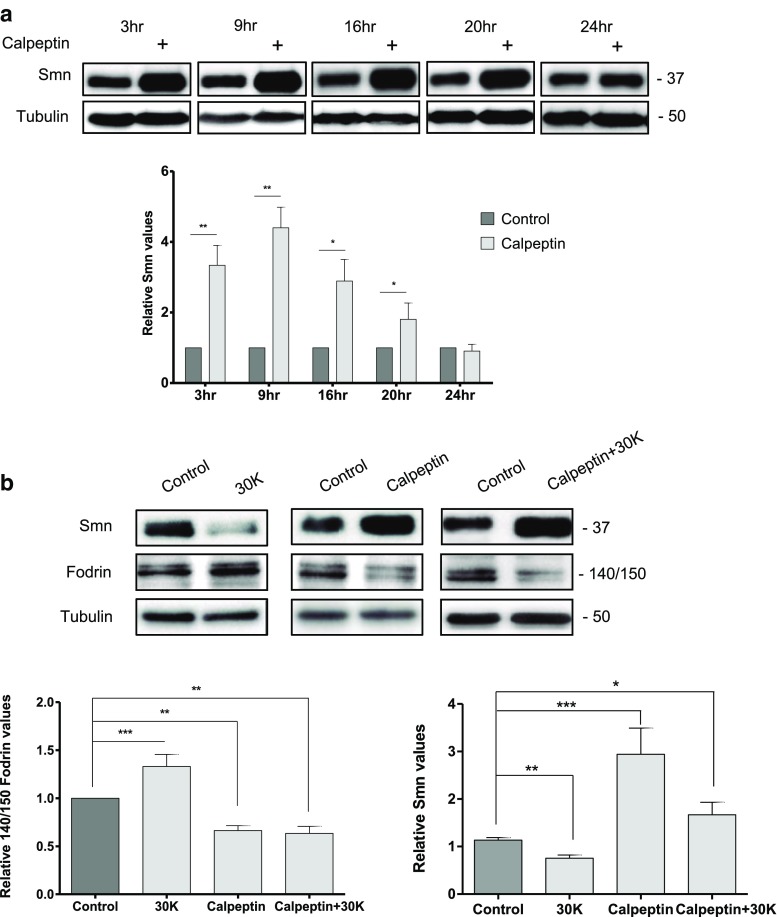
Calpeptin treatment increases Smn protein level in MNs. CD1 mouse MNs were isolated and cultured in the presence of NTFs. **a** Six days after plating, cells were treated with 25 μM calpeptin and cell lysates obtained 3, 9, 16, 20, and 24 h after treatment. Protein extracts were submitted to western blot analysis using an anti-SMN antibody. Membranes were reprobed with an anti-α-tubulin antibody. Graph values represent the expression of Smn vs α-tubulin and correspond to the quantification of five independent experiments ± SEM. Asterisks indicate significant differences using Student *t* test (*p < 0.05; **p < 0.005). **b** Six days after plating, cells were treated with 30 K or 25 μM calpeptin or 25 μM calpeptin+30K in the presence of NTFs. Three hours after treatment, cell lysates were obtained and submitted to western blot using anti-SMN or anti-α-fodrin antibodies. Membranes were reprobed with an anti-α-tubulin antibody. Graph values represent the expression of Smn (right) or 150/145 kDa fodrin product (left) vs α-tubulin and correspond to the quantification of at least four independent experiments ± SEM. Asterisks indicate significant differences using Student *t* test (*p < 0.05; **p < 0.001; ***p < 0.0001)

To further assess the role of calpain activity on Smn protein level in MNs, we used the cell-permeable inhibitor calpeptin [33]. We first analyzed the effect of calpeptin in basal culture conditions. E12.5 MNs were isolated and cultured in the presence of NTFs. Six days later, plating culture medium was changed to fresh medium containing NTFs or NTFs plus 25 μM calpeptin. Total cell lysates were obtained at 3, 9, 16, 20, and 24 h after treatment and submitted to western blot protein analysis using an anti-Smn antibody. Smn protein level was significantly increased after 3, 9, 16, and 20 h of calpeptin treatment, compared to non-treated controls (3.34 ± 0.56, *p* < 0.005; 4.40 ± 0.58, *p* < 0.005; 2.89 ± 0.61, *p* < 0.05; and 2.06 ± 0.50, *p* < 0.05, respectively; Fig. 3a). No changes of Smn protein were observed in cells treated 24 h, indicating that calpain inhibition using calpeptin increases Smn up to 24 h of treatment in cultured MNs. No changes in Smn protein were observed in cells treated 24 h with calpeptin, indicating that inhibition of calpain by calpeptin increased Smn up to 24 h of treatment in cultured MNs. This temporary effect may have been due to the degradation of calpeptin; the providers recommend that the aqueous solution not be stored for more than 1 day. Future research should consider the effect of calpeptin degradation by refreshing the media and/or varying other experimental conditions.

To study whether calpeptin treatment reverts Smn reduction caused by chronic membrane depolarization, primary CD1 MN cultures were established. Six days after plating, cells were treated with NTFs (control) or NTFs plus 30K or 25 μM calpeptin or 25 μM calpeptin+30K. Three hours after treatment, cell lysates were obtained and submitted to western blot using anti-α-fodrin or anti-SMN antibodies. Degradation of α-fodrin to the 150/145 kDa-specific fodrin breakdown products indicates calpain activation [34]. The levels of 150/145 kDa product in cells treated with NTFs plus 30K (30K) were significantly increased compared to NTF control condition (1.33 ± 0.13, *p* < 0.0001). However, the addition of calpeptin to NTF control or 30K conditions significantly reduced fodrin cleavage products (0.66 ± 0.05, *p* < 0.001, and 0.63 ± 0.07, *p* < 0.001, respectively) (Fig. 3b). This result suggests that calpain is activated after membrane depolarization in mouse MNs. Smn protein level in calpeptin+30K-treated cells (1.67 ± 0.26, *p* < 0.05) was significantly increased compared to 30K and control conditions (Fig. 3b). These results together indicate that Smn is reduced in 30K-treated cells, and this effect can be prevented by calpeptin treatment.

### High Potassium Treatment Induces Smn Cleavage in Cultured MNs

In muscle tissue and in U2-OS cell lines, SMN is processed by calpain leading to the production of N- and C- terminal cleavage products [24, 25]. To identify some of these cleavage products in cultured MNs, we isolated E12.3 MNs from CD1 mice and cultured for 6 days in the presence of NTFs. Cells were treated for 3 h with 30 mM KCl (30K) or with 25μM calpeptin or 30K plus 25 μM calpeptin, and total lysates were analyzed by western blot using two monoclonal antibodies, anti-SMN (Clone 8) (BD Bioscience) and anti-SMN1 antibody (9F2) (Cell Signaling), to detect the cleavage products. Anti-SMN antibody (Clone 8) recognizes full-length and N-terminal fragments, and anti-SMN1 (9F2) antibody recognizes C-terminal fragments. Quantification of full-length Smn (~ 37 kDa) showed that 30K treatment significantly reduced Smn protein level (0.59 ± 0.07, *p* < 0.0001), whereas calpeptin treatment increased Smn (2.27 ± 0.50, *p* < 0.05), compared to non-treated control. When the same membranes were overexposed, we detected an increase of N-terminal Smn cleavage product (~ 20 kDa) in 30K condition (1.98 ± 0.50, *p* < 0.05), compared to the untreated control, which was prevented by addition of 25 μM calpeptin to the medium (0.53 ± 0.12, *p* < 0.05) (data not shown).

**Fig. 4:**
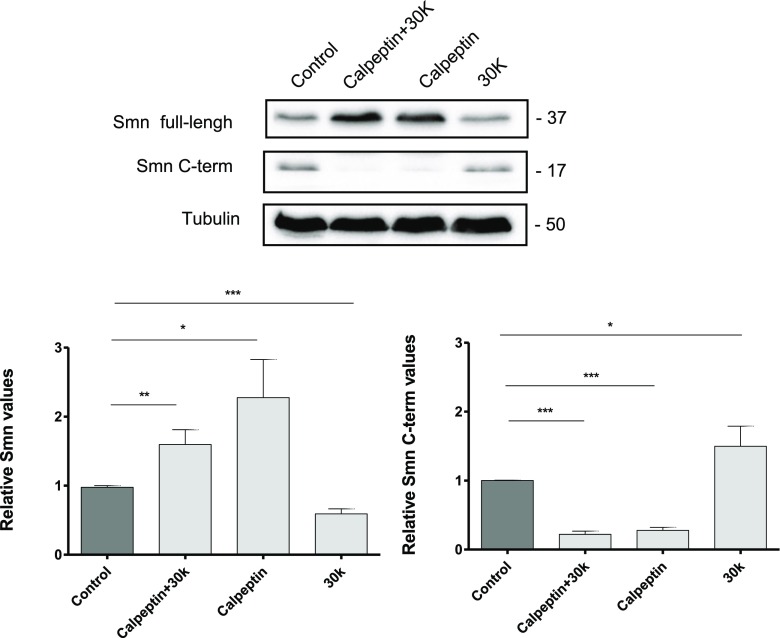
Effect of high K^+^ and calpeptin treatment on SMN cleavage. CD1 MNs were cultured in the presence of NTFs. Six days after plating, cells were treated with 30 mM KCl (30K) or 25 μM calpeptin or 30K plus 25 μM calpeptin. Three hours later, cell lysates were obtained and submitted to western blot using an anti-SMN (full length) or anti-SMN1 (C-terminal fragments) antibodies. Membranes were reprobed with an anti-α-tubulin antibody. Graph values represent the expression of full-length Smn (left) or C-terminal Smn fragments (right) vs α-tubulin and correspond to the quantification of at least three independent experiments ± SEM. Asterisks indicate significant differences using Student *t* test (*p < 0.05; **p < 0.005; ***p < 0.0001)

Using the anti-SMN1 antibody, C-terminal fragments (~ 17 kDa) were only evident when the membrane was overexposed. As shown in Fig. 4, Smn C-terminal fragments were significantly increased in 30K (1.49 ± 0.29; *p* < 0.05), compared to control. Conversely, calpeptin treatment significantly reduced C-terminal fragments in 30K and control conditions (0.22 ± 0.04 *p* < 0.05, and 0.27 ± 0.04, *p* < 0.0001, respectively). These results together suggest that high K^+^ treatment reduces full-length Smn and increases C-terminal fragments.

### Calpeptin Treatment Increases Smn Protein Level and Prevents Neurite Degeneration in SMA Mutant MNs

**Fig. 5:**
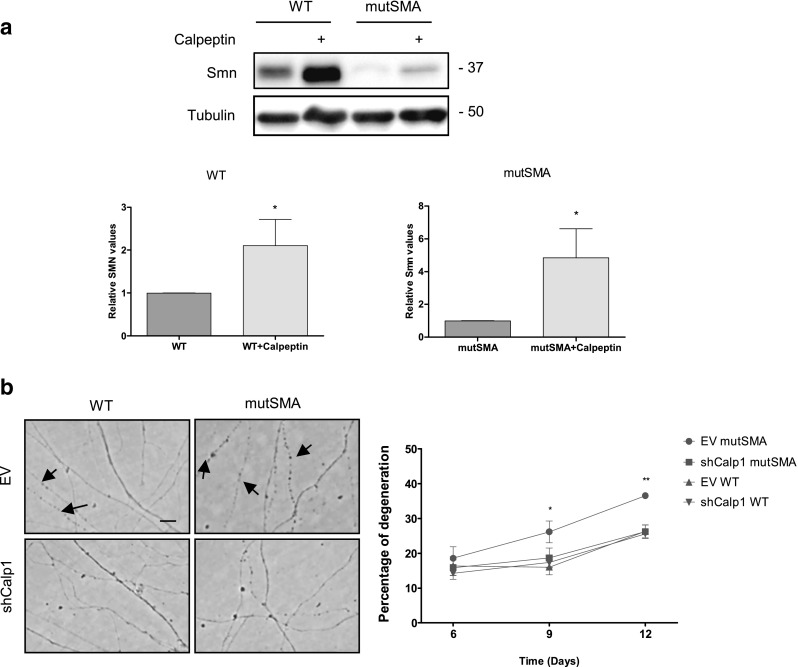
Calpeptin treatment increments Smn protein level and prevents neurite degeneration in SMA MNs. E12.5 isolated MNs from WT and mutSMA mice were cultured in the presence of NTFs. **a** Six days after plating, cells were treated with 25 μM calpeptin or left untreated. Three hours later, protein extracts were obtained and submitted to western blot analysis using an anti-SMN antibody. Membranes were reprobed with an anti-α-tubulin antibody. Graph values represent the expression of Smn vs α-tubulin in WT and mutSMA (left and right) and correspond to the quantification of three independent experiments ± SEM. Asterisks indicate significant differences using Student *t* test (*p < 0.05). **b** Low-density MN cultures of WT and mutSMA mice were established in the presence of NTFs and transduced with lentivirus containing the shCalp1 construct or the empty vector (EV) construct. Representative images of cells cultured for 9 days after transduction. Arrows indicate neurite degeneration. Scale bar 12 μm. The percentage of degenerating neurites was measured as described in “Material and Methods.” Graph values are the mean number of degenerating neurites per microscopic area for each condition of 12 wells in four independent experiments ± SEM. Asterisks indicate significant differences using two-way ANOVA test and Bonferroni posttest (*p < 0.01; ** *p* < 0.0005)

To determine whether calpeptin treatment regulates Smn in MNs from E12.5 embryos of the severe SMA transgenic mouse model (FVB·Cg-Tg (SMN2)89AhmbSmn1tm1Msd/J), mice were genotyped and the spinal cords of wild-type (WT) and mutant (mutSMA) were dissected. WT and mutSMA isolated MNs were cultured in the presence of NTFs, carefully following the same protocol used for CD1 cells: six days after plating, WT and mutSMA cells were treated with 25 μM calpeptin during 3 h. Protein extracts were collected and submitted to western blot analysis using anti-SMN antibody. Results indicate that Smn is increased in calpeptin-treated WT condition (2.13 ± 0.50-fold induction, *p* < 0.05) compared to WT control (Fig. 5a, left graph). Furthermore, in mutSMA cultures, calpeptin treatment increased Smn protein, compared to mutSMA non-treated cells (5.18 ± 1.48-fold induction, *p* < 0.05) (Fig. 5a, right graph).

Focal axonal swellings have been previously described in Smn-reduced mouse tissues and MNs [27, 35]. To determine the presence of neurite degeneration in MNs obtained from the severe SMA mouse model (Smn^−/−^; SMN2^+/+^), cells were isolated from the mutSMA and WT mice at E12.5 and cultured at a low density (~ 5000 cells/cm^2^). We assessed neurite degeneration by quantifying neurite segments in a determinate area of the plate using phase-contrast microscopy at days 6, 9, and 12. Neurites were considered degenerated if they showed evidence of swellings and/or blebbing (see “Materials and Methods”) [30]. Nine and 12 days after plating in the presence of NTFs, mutSMA MNs showed a higher percentage of degeneration compared to WT MNs (9 days, WT 15.68 ± 6.67% of degenerated neurites and mutSMA 30.13 ± 13.10%, *p* < 0.05; 12 days, WT 24.10 ± 7.37% and mutSMA 43.08 ± 4.71%, *p* < 0.01) (data not shown). In order to analyze the effect of calpain reduction on neurite degeneration, mutSMA and WT cells were transduced with the lentivirus carrying shCalp1 or EV constructs. After 6 days of transduction, no significant differences were observed between groups. However, 9 days after transduction, we detected a significant difference in neurite morphology when the shCalp1 mutSMA (16.64 ± 5.67%) was compared with the EV mutSMA (26.17 ± 8.22%, *p* < 0.01) cultures. After 12 days, the signs of degeneration increased to 36.55 ± 2.28% in EV mutSMA cultures, whereas shCalp1 mutSMA cultures showed significantly reduced degeneration (29.24 ± 4.89%, *p* < 0.0005), compared to the EV mutSMA control (Fig. 5b). This percentage did not differ from those observed in EV WT (26.29 ± 4.69%) and shCalp1 WT (25.62 ± 3.61%). All these results together demonstrate that calpain regulates Smn protein level and neurite degeneration in Smn-reduced MNs.

### Calpeptin Administration Extends Survival of Severe SMA and SMNdelta7 SMA Mice

**Fig. 6:**
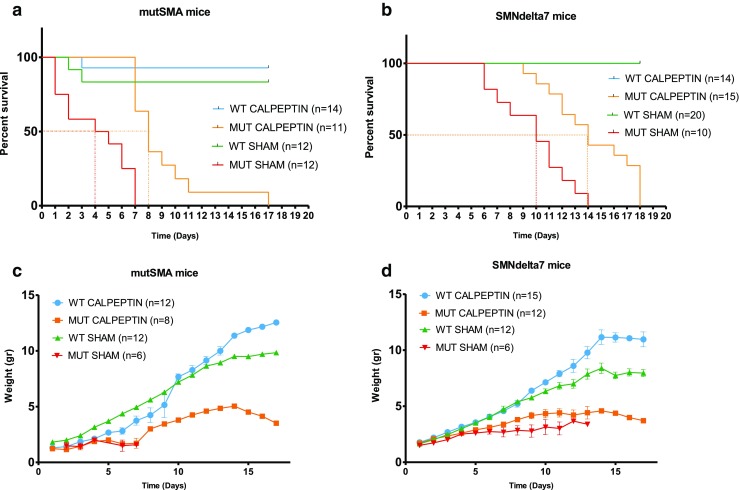
Calpeptin administration extends survival of severe SMA and SMNdelta7 SMA mice. **a** Survival of mutSMA treated with calpeptin. **b** Survival of SMNdelta7 treated with calpeptin. The difference in survival between calpeptin-treated mutSMA and SMNdelta7 mice and non-treated was calculated by the log-rank Mantel-Cox test (*p* < 0.0001). Weight gain of mutSMA (**c**) and SMNdelta7 (**d**) mice treated with calpeptin. Error bars indicate SEM

The evidences suggested that calpain regulates Smn protein level positively in MNs. Therefore, we decided to examine whether calpeptin administration had a positive effect on SMA mice. To further assess this hypothesis, we started a treatment protocol using two different SMA mouse models: the FVB·Cg-Tg (SMN2)89AhmbSmn1tm1Msd/J (mutSMA) and the FVB.Cg-Grm7Tg(SMN2)89Ahmb Smn1tm1Msd Tg (SMN2*delta7)4299Ahmb/J (SMNdelta7). To determine if calpeptin affected the lifespan and the body weight of these mice, animals were tattooed and genotyped at postnatal day 0 (P0). WT and mutants littermates were grouped by sham or treatment and the subcutaneous injections were started at P1, with a daily dose of 6 μg calpeptin per gram of weight. Results showed that calpeptin administration significantly improved lifespan of the mutSMA-calpeptin group (average days 9 ± 2.97, *n* = 11), compared with the mutSMA-sham (average days 4.08 ± 2.54, *n* = 12, *p* < 0.0001) (Fig. 6a). Likewise, lifespan of SMNdelta7-calpeptin group (average days 14.41 ± 2.90, *n* = 15) was significantly increased compared to SMNdelta7-sham (average days 9.8 ± 2.89, *n* = 10, *p* < 0.0001) mice (Fig. 6b).

When the weight of the animal groups was measured, we did not find significant differences in calpeptin-treated mutant compared to non-treated mice. In contrast, weight was slightly increased in calpeptin-treated WT groups compared with sham-treated WT groups from day 13 of treatment to the end of the experiment (Fig. 6c, d).

### Effect of Calpeptin Treatment on Motor Function in SMA Mice

**Fig. 7:**
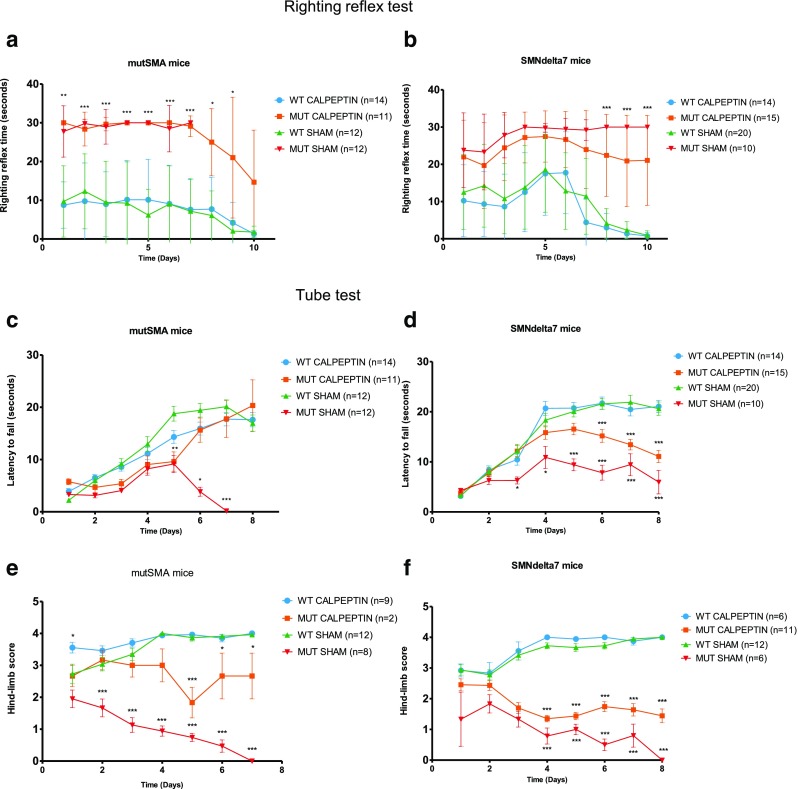
Calpeptin treatment improves motor function in SMA mice. Mean time to right ± SEM between P1 and P10 measured in mutSMA groups (**a**) or SMNdelta7 groups (**b**) Two-way ANOVA test and Bonferroni posttest were performed to compare calpeptin-treated and untreated groups. Asterisks indicate significant differences (*p < 0.05; ** *p* < 0.01; *** *p* < 0.0001). Mean of latency to fall ± SEM between P2 and P8 was measured in mutSMA groups (**c**) and SMNdelta7 groups (**d**), and mean of hind-limb score ± SEM between P2 and P8 was measured in mutSMA groups (**e**) and SMNdelta7 groups (**f**). Two-way ANOVA test and Bonferroni posttest was performed to compare calpeptin-treated and untreated groups. Asterisks indicate significant differences (*p < 0.05; ** *p* < 0.01; *** *p* < 0.001)

Because mutSMA and SMNdelta7 mouse models are characterized by severe motor function impairment, we analyzed whether the enhancement in lifespan observed in both models after calpeptin treatment was correlated with motor functional improvement. Two behavior tests were performed: the righting reflex test and the tube test [32, 36]. The righting reflex response is based on the ability of neonatal mice to return to their four paws after having been placed in supine position. It can be measured in pups as early as P1-P2 and evaluated until P9-P10. The test was performed on mice in the sham- or calpeptin-treated WT and sham- or calpeptin-treated mutant (mutSMA and SMNdelta7) groups. The test was designed with a maximum time of 30 s and measurements were taken daily before the calpeptin injection from P1 to P10, done in triplicate for each animal with 5 min resting time between tests. Results obtained in WT groups showed that the righting reflex time was consistently faster (~ 10 s) than in mutant groups (~ 30 s). In calpeptin-treated mutSMA, there were no significant differences from the beginning of the treatment (P1) to P7 compared to sham-treated mutSMA. However, from P7 to the end of the experiment the righting reflex time progressively decreased in calpeptin-treated mutSMA and SMNdelta7 (~ 20 s) (Fig. 7a, b).

The tube test is a non-invasive motor function test specifically designed for neonatal rodents. It evaluates the proximal hind-limb muscle strength, weakness, and fatigue. The animal age range for this test is from P2 to P8. Two parameters were evaluated in the present study: latency to fall from the edge of the tube (in seconds, latency to fall graphs) and the hind-limb score (HLS graphs) which assess the positioning of the legs and tail. Mice with motor weakness show reduced time to fall and low score of HLS. The test was performed in the same sham and treated groups of mice described. The test was designed with a maximum time of 30 s, and measures were performed in triplicate daily before the calpeptin injection from P1 to P8. When the latency time was analyzed in the mutSMA model, we observed no differences in time-to-fall from P1 to P5. Nevertheless, on days 6 (P6) and 7 (P7) after treatment, calpeptin-treated mutSMA mice (P6, 15.62 ± 10.66 s, *n* = 7) showed significantly increased time-to-fall compared to sham-treated mutSMA group (P6, 3.84 ± 1.92, *n* = 3, *p* < 0.05) and no significant differences compared to WT groups (P6 WT-SHAM, 19.4 ± 9.5) (Fig. 7c). In mutant SMNdelta7 mice, calpeptin treatment significantly increased latency-to-fall compared to sham-treated controls from P3 to the end of the experiment (*p* < 0.05) (Fig. 7d). In both SMA models, HLS measures revealed that calpeptin treatment of mutant mice significantly increased the score compared to sham mutants (mutSMA P2 to P8, *p* < 0.001; SMNdelta7 P6 to P8, *p* < 0.05) (Fig. 7e, f). Altogether, the results obtained from the behavior tests suggested that calpeptin treatment ameliorates motor function of mutant mice in both SMA models analyzed.

## Discussion

In the present study, calpain regulated Smn protein level in a primary culture of isolated CD1 and Smn-reduced mice spinal cord MNs. Calpain 1 knockdown or calpeptin treatment increased Smn in these cells. Subcutaneous administration of the calpain inhibitor, calpeptin, improved the survival and phenotype of severe and SMNdelta7 SMA mice. In agreement with previous publications demonstrating that Smn located in the muscle cell is a proteolytic target of calpain [24, 25], we showed here that Smn protein level in MNs can be modulated by calpain. However, the functional implications of this cleavage are not completely understood in the muscle cells and neurons. SMN protein can be processed by caspases and calpain. It has been previously reported that SMN calpain cleavage sites are distinct from caspase sites. Apoptosis activity produces an N-terminal SMN product [37], whereas western analysis of calpain assays detects two SMN cleavage products (N- and C-terminus) [25]. In our MN culture system, membrane depolarization treatment clearly increased C-terminal Smn level, which is prevented by calpain inhibition. One of the options to explain the importance of SMN cleavage in the cellular function can be related to the role of the proteolytic isoforms in cytoskeletal dynamics. In this context, many evidences establish the importance of actin cytoskeletal dynamics in SMN function and SMA pathology [38]. Moreover, it has been described that the C-terminal domain of SMN can regulate the G- to F-actin ratio, and the expression of this terminal fragment can rescue neurite outgrowth defects in PC12 cells that have been depleted of endogenous Smn [39]. In cultured CD1 MNs, we have observed that calpeptin treatment reduces Smn C-terminal fragment and increases full-length Smn, indicating that calpain basal activity may process Smn and regulate its level in these cells. Together, the muscle and MN results strongly suggest a significant role of calpain activity in SMN protein level control and SMA. Calpain is a regulatory protease, whose action typically activates or inactivates a given protein target. Its activity is compartmentalized and works in microenvironments where calcium can be controlled [18]. In this context, it can be postulated that calcium changes localized in the axonal compartment described in SMA [40, 41] may contribute to the deregulation of calpain activation and SMN processing.

The calcium/calpain pathway also controls the autophagy process, which can be regulated by changes in intracellular calcium level. It has been reported that pharmacological inhibition of calpain or its genetic knockdown increases autophagic flux without perturbing mTORC1 [42]. Pharmacological release of calcium from intracellular stores blocks autophagic flux, thereby increasing the number of autophagosomes, and retards autophagic cargo clearance. In contrast, influx of extracellular calcium through L-type calcium channel agonists inhibits autophagy at the level of autophagosome synthesis [43]. Autophagy has been suggested as a fundamental process whose dysregulation can be involved in neurodegenerative disorders and might provide a potential target for therapy. In fact, SMA mice treated with the autophagy inhibitor 3-methyladenine showed delayed MN degeneration, improved motor behavior, and increased lifespan of SMA [44] and the neuroprotective effects of TTC (non-toxic carboxy-terminal fragment of tetanus toxin heavy chain) treatment in SMA mice are related to the reduced expression of autophagy markers [45]. Previous results in cultured MNs, reported in Periyakaruppiah et al. (2016), show that shCalp1 treatment increased the level of LC3 II, suggesting that the endogenous reduction of calpain increases autophagosome number. These results are in accordance with previous reports that overexpression of constitutively active calpain inhibits autophagosome synthesis and that calpeptin treatment increases the number of autophagosome-like structures and decreases the number of mitochondria (which are endogenous autophagy substrates) in COS-7 cells [42]. In the present study, calpeptin treatment ameliorates survival and phenotype in two SMA mouse models. These observations, together with the autophagy deregulation observed in SMN-reduced cells [15, 16, 46], support the hypothesis that autophagy manipulation may be useful in treating SMA, always keeping in mind the complexity of the autophagy process and avoiding to attribute the results obtained exclusively to autophagy regulation. In this context, future studies focused on the role of proteins involved in autophagy and other intracellular SMA-altered mechanisms are needed to identify potential targets for combinatorial therapeutics. Several mechanisms have positive effects on SMA phenotype and should be regarded as candidates for these combinatorial approaches, including autophagy pathway modulators such as the autophagy inhibitor 3-methyladenine [44] or the inhibition of the autophagy adaptor p62 [47], butyrate-based compounds and other inhibitors of histone deacetylase activity [11], and regulators of the calcium-dependent acting-bundling protein Plastin-3 [48]. Other regulatory mechanisms involved in the degradation of SMN, such as the ubiquitin-proteasome system, also warrant analysis. The inhibition of both proteasome and calpain may contribute to our understanding of these pathways to SMN protein degradation.

Calpain inhibition or reduction clearly regulates Smn level in MNs, prevents neurite degeneration, and improves survival and phenotype of SMA mice models. However, calpeptin treatment does not completely reverse the disease phenotype observed in these mice. SMA disease complexity is likely related to several functions of SMN which give rise to the complete SMA pathology. For example, the molecular contribution of SMN reduction in muscle and satellite cells of skeletal muscle on SMA phenotype remains unclear. It is known that calpain regulates the level of SMN in this tissue; consequently, it may be suggested that calpeptin treatment would also ameliorate SMA phenotype due to increased SMN in these cells. Nevertheless, further histopathologic analysis of skeletal muscle would help to clarify this hypothesis.

SMA therapeutic approaches are designed as “SMN dependent,” contributing to increased SMN levels or “SMN independent,” which enhance the SMN-associated functions or compensate for a secondary defect. We provide here a “SMN dependent” target for SMA therapeutics: calpain inhibition. Our in vitro experiments showed that calpeptin treatment or calpain 1 knockdown can increase SMN protein in MNs. This effect is achieved by inhibiting calpain and increasing SMN protein stability in these cells. Moreover, preliminary data using spinal cord protein extracts from calpeptin-treated wild-type and mutant delta7 mice suggest an increase in Smn, compared to controls (data not shown). In the context of our ongoing research, we propose that quantification of calpain expression and activity in SMA human tissue would be of value for understanding the involvement of this protease in SMN-reduced MN degeneration.

Calpain activation has been implicated in several neurological diseases, including amyotrophic lateral sclerosis, Alzheimer disease, Parkinson disease, stroke, and brain trauma. Inhibitors of calpain have possible therapeutic applications to a range of disorders with neuronal cell loss. For example, calpain inhibition is beneficial to the ALS phenotype [49] and several clinical trials are underway with compounds that inhibit calpain in Alzheimer disease [50]. Some authors suggest a possible common neurodegenerative mechanism shared by several adult onset brain disorders. Here, we demonstrated that calpain inhibition is also beneficial in ameliorating a childhood-onset disease, which focuses the relevance of calpain activity as an essential mechanism regulating neurodegeneration in diseases of different origin.

## References

[1] Lunn MR, Wang CH (2008). Spinal muscular atrophy. Lancet.

[2] Tisdale S, Pellizzoni L (2015). Disease mechanisms and therapeutic approaches in spinal muscular atrophy. J Neurosci.

[3] Sumner CJ (2006). Therapeutics development for spinal muscular atrophy. NeuroRx.

[4] Crawford TO, Pardo CA (1996). The neurobiology of childhood spinal muscular atrophy. Neurobiol Dis.

[5] Lefebvre S, Burglen L, Reboullet S, Clermont O, Burlet P, Viollet L, Benichou B, Cruaud C, Millasseau P, Zeviani M (1995). Identification and characterization of a spinal muscular atrophy-determining gene. Cell.

[6] Lorson CL, Hahnen E, Androphy EJ, Wirth B (1999). A single nucleotide in the SMN gene regulates splicing and is responsible for spinal muscular atrophy. Proc Natl Acad Sci U S A.

[7] Monani UR, Lorson CL, Parsons DW, Prior TW, Androphy EJ, Burghes AH, McPherson JD (1999). A single nucleotide difference that alters splicing patterns distinguishes the SMA gene SMN1 from the copy gene SMN2. Hum Mol Genet.

[8] Lefebvre S, Burlet P, Liu Q, Bertrandy S, Clermont O, Munnich A, Dreyfuss G, Melki J (1997). Correlation between severity and SMN protein level in spinal muscular atrophy. Nat Genet.

[9] Finkel RS, Mercuri E, Darras BT, Connolly AM, Kuntz NL, Kirschner J, Chiriboga CA, Saito K, Servais L, Tizzano E, Topaloglu H, Tulinius M, Montes J, Glanzman AM, Bishop K, Zhong ZJ, Gheuens S, Bennett CF, Schneider E, Farwell W, de Vivo DC, ENDEAR Study Group (2017). Nusinersen versus sham control in infantile-onset spinal muscular atrophy. N Engl J Med.

[10] Wirth B, Barkats M, Martinat C, Sendtner M, Gillingwater TH (2015). Moving towards treatments for spinal muscular atrophy: hopes and limits. Expert Opin Emerg Drugs.

[11] Butchbach ME, Lumpkin CJ, Harris AW, Saieva L, Edwards JD, Workman E, Simard LR, Pellizzoni L, Burghes AH (2016). Protective effects of butyrate-based compounds on a mouse model for spinal muscular atrophy. Exp Neurol.

[12] Kwon DY, Motley WW, Fischbeck KH, Burnett BG (2011). Increasing expression and decreasing degradation of SMN ameliorate the spinal muscular atrophy phenotype in mice. Hum Mol Genet.

[13] Burnett BG, Muñoz E, Tandon A, Kwon DY, Sumner CJ, Fischbeck KH (2009). Regulation of SMN protein stability. Mol Cell Biol.

[14] Kernochan LE, Russo ML, Woodling NS, Huynh TN, Avila AM, Fischbeck KH, Sumner CJ (2005). The role of histone acetylation in SMN gene expression. Hum Mol Genet.

[15] Periyakaruppiah A, de la Fuente S, Arumugam S, Bahi N, Garcera A, Soler RM (2016). Autophagy modulators regulate survival motor neuron protein stability in motoneurons. Exp Neurol.

[16] Garcera A, Bahi N, Periyakaruppiah A, Arumugam S, Soler RM (2013). Survival motor neuron protein reduction deregulates autophagy in spinal cord motoneurons in vitro. Cell Death Dis.

[17] Foran E, Kwon DY, Nofziger JH, Arnold ES, Hall MD, Fischbeck KH, Burnett BG (2016). CNS uptake of bortezomib is enhanced by P-glycoprotein inhibition: implications for spinal muscular atrophy. Neurobiol Dis.

[18] Goll DE, Thompson VF, Li H, Wei W, Cong J (2003). The calpain system. Physiol Rev.

[19] Yamashita T, Hideyama T, Hachiga K, Teramoto S, Takano J, Iwata N, Saido TC, Kwak S (2012). A role for calpain-dependent cleavage of TDP-43 in amyotrophic lateral sclerosis pathology. Nat Commun.

[20] Shields DC, Schaecher KE, Hogan EL, Banik NL (2000). Calpain activity and expression increased in activated glial and inflammatory cells in penumbra of spinal cord injury lesion. J Neurosci Res.

[21] Yamashima T (2013). Reconsider Alzheimer's disease by the ‘calpain-cathepsin hypothesis’—a perspective review. Prog Neurobiol.

[22] Cowan CM, Fan MM, Fan J, Shehadeh J, Zhang LY, Graham RK, Hayden MR, Raymond LA (2008). Polyglutamine-modulated striatal calpain activity in YAC transgenic Huntington disease mouse model: impact on NMDA receptor function and toxicity. J Neurosci.

[23] Vosler PS, Brennan CS, Chen J (2008). Calpain-mediated signaling mechanisms in neuronal injury and neurodegeneration. Mol Neurobiol.

[24] Walker MP, Rajendra TK, Saieva L, Fuentes JL, Pellizzoni L, Matera AG (2008). SMN complex localizes to the sarcomeric Z-disc and is a proteolytic target of calpain. Hum Mol Genet.

[25] Fuentes JL, Strayer MS, Matera AG (2010). Molecular determinants of survival motor neuron (SMN) protein cleavage by the calcium-activated protease, calpain. PLoS One.

[26] Gou-Fabregas M, Garcera A, Mincheva S, Perez-Garcia MJ, Comella JX, Soler RM (2009). Specific vulnerability of mouse spinal cord motoneurons to membrane depolarization. J Neurochem.

[27] Garcera A, Mincheva S, Gou-Fabregas M, Caraballo-Miralles V, Llado J, Comella JX, Soler RM (2011). A new model to study spinal muscular atrophy: Neurite degeneration and cell death is counteracted by BCL-X-L overexpression in motoneurons. Neurobiol Dis.

[28] Soler RM, Egea J, Mintenig GM, Sanz-Rodriguez C, Iglesias M, Comella JX (1998). Calmodulin is involved in membrane depolarization-mediated survival of motoneurons by phosphatidylinositol-3 kinase- and MAPK-independent pathways. J Neurosci.

[29] Arce V, Garces A, de Bovis B, Filippi P, Henderson C, Pettmann B, deLapeyriere O (1999). Cardiotrophin-1 requires LIFRbeta to promote survival of mouse motoneurons purified by a novel technique. J Neurosci Res.

[30] Press C, Milbrandt J (2008). Nmnat delays axonal degeneration caused by mitochondrial and oxidative stress. J Neurosci.

[31] Schneider CA, Rasband WS, Eliceiri KW (2012). NIH Image to ImageJ: 25 years of image analysis. Nat Methods.

[32] El-Khodor BF, Edgar N, Chen A, Winberg ML, Joyce C, Brunner D, Suárez-Fariñas M, Heyes MP (2008). Identification of a battery of tests for drug candidate evaluation in the SMNDelta7 neonate model of spinal muscular atrophy. Exp Neurol.

[33] Adamec E, Beermann ML, Nixon RA (1998). Calpain I activation in rat hippocampal neurons in culture is NMDA receptor selective and not essential for excitotoxic cell death. Brain Res Mol Brain Res.

[34] Nath R, Raser KJ, Stafford D, Hajimohammadreza I, Posner A, Allen H, Talanian RV, Yuen P, Gilbertsen RB, Wang KK (1996). Non-erythroid alpha-spectrin breakdown by calpain and interleukin 1 beta-converting-enzyme-like protease(s) in apoptotic cells: contributory roles of both protease families in neuronal apoptosis. Biochem J.

[35] McGovern VL, Beattie CE, Burghes AH, Gavrilina TO (2008). Embryonic motor axon development in the severe SMA mouse. Hum Mol Genet.

[36] Butchbach ME, Edwards JD, Burghes AH (2007). Abnormal motor phenotype in the SMNDelta7 mouse model of spinal muscular atrophy. Neurobiol Dis.

[37] Kerr DA, Nery JP, Traystman RJ, Chau BN, Hardwick JM (2000). Survival motor neuron protein modulates neuron-specific apoptosis. Proc Natl Acad Sci U S A.

[38] Oprea GE, Kröber S, McWhorter ML, Rossoll W, Müller S, Krawczak M, Bassell GJ, Beattie CE, Wirth B (2008). Plastin 3 is a protective modifier of autosomal recessive spinal muscular atrophy. Science.

[39] van Bergeijk J, Rydel-Könecke K, Grothe C, Claus P (2007). The spinal muscular atrophy gene product regulates neurite outgrowth: importance of the C terminus. FASEB J.

[40] Ruiz R, Casañas JJ, Torres-Benito L, Cano R, Tabares L (2010). Altered intracellular Ca2+ homeostasis in nerve terminals of severe spinal muscular atrophy mice. J Neurosci.

[41] Jablonka S, Beck M, Lechner BD, Mayer C, Sendtner M (2007). Defective Ca2+ channel clustering in axon terminals disturbs excitability in motoneurons in spinal muscular atrophy. J Cell Biol.

[42] Williams A, Sarkar S, Cuddon P, Ttofi EK, Saiki S, Siddiqi FH, Jahreiss L, Fleming A, Pask D, Goldsmith P, O'Kane CJ, Floto RA, Rubinsztein DC (2008). Novel targets for Huntington's disease in an mTOR-independent autophagy pathway. Nat Chem Biol.

[43] Sarkar S (2013). Regulation of autophagy by mTOR-dependent and mTOR-independent pathways: autophagy dysfunction in neurodegenerative diseases and therapeutic application of autophagy enhancers. Biochem Soc Trans.

[44] Piras A, Schiaffino L, Boido M, Valsecchi V, Guglielmotto M, De Amicis E, Puyal J, Garcera A, Tamagno E, Soler RM, Vercelli A (2017). Inhibition of autophagy delays motoneuron degeneration and extends lifespan in a mouse model of spinal muscular atrophy. Cell Death Dis.

[45] Oliván S, Calvo AC, Rando A, Herrando-Grabulosa M, Manzano R, Zaragoza P, Tizzano EF, Aquilera J, Osta R (2016). Neuroprotective effect of non-viral gene therapy treatment based on tetanus toxin C-fragment in a severe mouse model of spinal muscular atrophy. Front Mol Neurosci.

[46] Custer SK, Androphy EJ (2014). Autophagy dysregulation in cell culture and animals models of spinal muscular atrophy. Mol Cell Neurosci.

[47] Rodriguez-Muela N, Parkhitko A, Grass T, Gibbs RM, Norabuena EM, Perrimon N, Singh R, Rubin LL (2018). Blocking p62-dependent SMN degradation ameliorates spinal muscular atrophy disease phenotypes. J Clin Invest.

[48] Kaifer KA, Villalón E, Osman EY, Glascock JJ, Arnold LL, Cornelison DD, Lorson CL (2017). Plastin-3 extends survival and reduces severity in mouse models of spinal muscular atrophy. JCI Insight.

[49] Rao MV, Campbell J, Palaniappan A, Kumar A, Nixon RA (2016). Calpastatin inhibits motor neuron death and increases survival of hSOD1(G93A) mice. J Neurochem.

[50] Wright AL, Vissel B (2016). CAST your vote: is calpain inhibition the answer to ALS?. J Neurochem.

